# Poly[[hexa­aqua­bis­(μ_3_-benzene-1,3,5-tricarboxyl­ato-κ^3^ *O* ^1^:*O* ^3^:*O* ^5^)bis­(5,5′-dimethyl-2,2′-bipyridine-κ^2^ *N*,*N*′)trizinc] hexa­hydrate]

**DOI:** 10.1107/S1600536811022173

**Published:** 2011-06-18

**Authors:** Wen-Wen Shan, Han-Lin Xiong, Chong-Zhen Mei

**Affiliations:** a North China University of Water Conservancy and Electric Power, Zhengzhou 450011, People’s Republic of China

## Abstract

In the title compound, {[Zn_3_(C_9_H_3_O_6_)_2_(C_12_H_12_N_2_)_2_(H_2_O)_6_]·6H_2_O}_*n*_, one Zn^II^ atom, lying on an inversion center, is six-coordinated by two O atoms from two benzene-1,3,5-tricarboxyl­ate (btc) ligands and four water mol­ecules in a distorted octa­hedral geometry. The other Zn^II^ atom is five-coordinated by two N atoms from a 5,5′-dimethyl-2,2′-bipyridine (dmbpy) ligand, two O atoms from two btc ligands and one water mol­ecule in a distorted trigonal–bipyramidal geometry. The compound features a one-dimensional ladder structure, with windows of *ca* 10.245 (1) × 15.446 (2) Å. The ladders are linked together by inter­molecular O—H⋯O hydrogen bonds and π–π inter­actions between the benzene rings and between the pyridine rings [centroid-to-centroid distances 3.858 (2) and 3.911 (3) Å, respectively] to form a three-dimensional supra­molecular structure. One of the lattice water molecules is disordered over two positions in a 0.592:0.408 ratio.

## Related literature

For background to network topologies and the applications of coordination polymers, see: Maspoch *et al.* (2007 ▶); Ockwig *et al.* (2005 ▶). For background to effective methods for the construction of coordination polymers, see: Du *et al.* (2007 ▶); Zang *et al.* (2006 ▶, 2010 ▶). For O—H⋯O hydrogen bonds, see: Desiraju (2004 ▶).
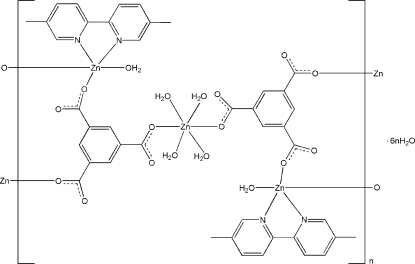


## Experimental

### 

#### Crystal data

[Zn_3_(C_9_H_3_O_6_)_2_(C_12_H_12_N_2_)_2_(H_2_O)_6_]·6H_2_O
*M* *_r_* = 1195.00
Triclinic, 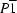

*a* = 10.2454 (10) Å
*b* = 10.5799 (10) Å
*c* = 12.658 (1) Å
α = 68.910 (8)°
β = 74.848 (8)°
γ = 81.834 (8)°
*V* = 1233.90 (19) Å^3^
*Z* = 1
Mo *K*α radiation
μ = 1.54 mm^−1^
*T* = 296 K
0.21 × 0.20 × 0.19 mm

#### Data collection

Bruker APEXII CCD diffractometer
Absorption correction: multi-scan (*SADABS*; Bruker, 2001 ▶) *T*_min_ = 0.739, *T*_max_ = 0.759
9149 measured reflections
4330 independent reflections
3426 reflections with *I* > 2σ(*I*)
*R*_int_ = 0.034

#### Refinement

*R*[*F* ^2^ > 2σ(*F* ^2^)] = 0.045
*wR*(*F* ^2^) = 0.124
*S* = 1.01
4330 reflections
336 parameters
12 restraints
H-atom parameters constrained
Δρ_max_ = 0.74 e Å^−3^
Δρ_min_ = −0.59 e Å^−3^

### 

Data collection: *APEX2* (Bruker, 2007 ▶); cell refinement: *SAINT* (Bruker, 2007 ▶); data reduction: *SAINT*; program(s) used to solve structure: *SHELXS97* (Sheldrick, 2008 ▶); program(s) used to refine structure: *SHELXL97* (Sheldrick, 2008 ▶); molecular graphics: *DIAMOND* (Brandenburg, 1999 ▶); software used to prepare material for publication: *SHELXTL* (Sheldrick, 2008 ▶).

## Supplementary Material

Crystal structure: contains datablock(s) I, global. DOI: 10.1107/S1600536811022173/hy2439sup1.cif

Structure factors: contains datablock(s) I. DOI: 10.1107/S1600536811022173/hy2439Isup2.hkl

Additional supplementary materials:  crystallographic information; 3D view; checkCIF report

## Figures and Tables

**Table 1:** Hydrogen-bond geometry (Å, °)

*D*—H⋯*A*	*D*—H	H⋯*A*	*D*⋯*A*	*D*—H⋯*A*
O1*W*—H1*WA*⋯O5^i^	0.84	1.93	2.754 (4)	166
O1*W*—H1*WB*⋯O6*W*^ii^	0.84	1.96	2.805 (5)	176
O2*W*—H2*WA*⋯O2^iii^	0.85	1.95	2.768 (4)	162
O2*W*—H2*WB*⋯O5*W*^iii^	0.85	2.14	2.806 (6)	135
O3*W*—H3*WB*⋯O3^iii^	0.85	2.16	2.740 (4)	125
O3*W*—H3*WC*⋯O5	0.85	2.26	2.705 (4)	113
O4*W*—H4*WA*⋯O5*W*	0.95	2.22	3.166 (19)	173
O4*W*′—H4*WD*⋯O2	0.87	2.63	3.499 (11)	177
O5*W*—H5*WA*⋯O6*W*	0.85	2.14	2.987 (9)	179
O5*W*—H5*WC*⋯O6^iv^	0.90	2.28	3.151 (7)	164
O6*W*—H6*WB*⋯O1^iv^	0.85	2.16	2.947 (5)	154
O6*W*—H6*WD*⋯O4^v^	0.85	2.23	3.019 (6)	154

Symmetry codes: (i) 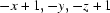; (ii) 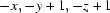; (iii) 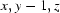; (iv) 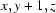; (v) 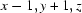.

**Fig. 1.**
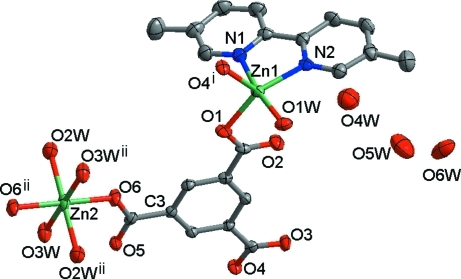
The asymmetric unit of the title compound. Displacement ellipsoids are drawn at the 50% probability level. H atoms have been omitted for clarity. [Symmetry codes: (i) x - 1, y, z; (ii) -x + 1, -y - 1, -z + 1.]

**Fig. 2.**
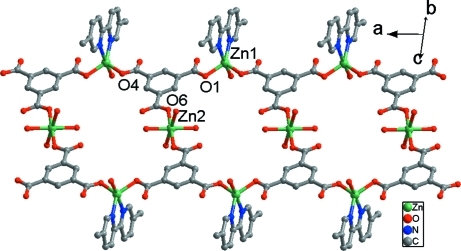
The one-dimensional ladder structure in the title compound.

**Fig. 3.**
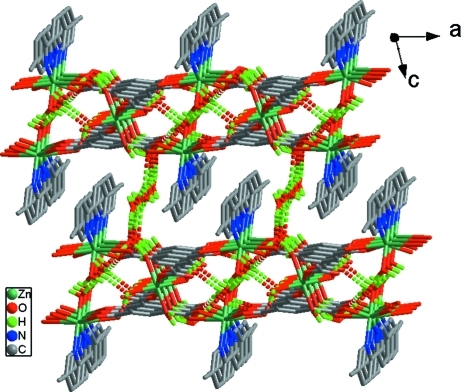
A view of the three-dimensional supramolecular structure in the title compound. Dashed lines represent hydrogen bonds.

## supplementary crystallographic information

### Comment

In recent years, supramolecular coordination assemblies have received much attention not only for their variety of architectures but also for the potential applications as functional materials (Maspoch *et al.*, 2007; Ockwig *et al.*, 2005). According to literature, carboxylate-based ligands are good bridging ligands to construct coordination polymers, in which many supramolecular structures have been furnished (Zang *et al.*, 2006, 2010). The rational assembly of target metal–organic networks depends on deliberate designs of the ligands with adjustable connectivity and a reasonable choice of metal ions with specific coordination nature. Additionally, the use of auxiliary ligands is also an effective method for the construction of coordination polymers (Du *et al.*, 2007). To further explore the influence of multicarboxylates and *N*-donor ligands on the properties and construction of coordination compounds, we undertake synthetic and structural studies on the title compound, a Zn(II) complex based on benzene-1,3,5-tricarboxylic acid (H_3_btc) and 5,5'-dimethyl-2,2'-bipyridine (dmbpy).

As shown in Fig. 1, the asymmetric unit of the title compound consists of one and a half Zn^II^ atoms, one btc ligand, one dmbpy ligand, three coordinated and three uncoordinated water molecules. Zn2 atom is located on an inversion center. Zn1 atom is coordinated by two O atoms from two btc ligands, one water molecule and two N atoms from one chelating dmbpy ligand, completing a distorted trigonal–bipyramidal geometry. N2, O1 and O4^i^ [symmetry code: (i) *x* - 1, *y*, *z*] comprise the equatorial plane, while O1W and N1 occupy the axial positions. Zn2 atom is in a distorted octahedral coordination environment and coordinated by two O atoms from a pair of symmetry-related btc ligands and four O atoms from two pairs of coordinated water molecules. O2W, O2W^ii^, O3W and O3W^ii^ [symmetry code: (ii) -*x* + 1, -*y* - 1, -*z* + 1] comprise the equatorial plane, while O6 and O6^ii^ occupy the axial positions. As depicted in Fig. 2, adjacent Zn1 atoms are linked together through btc ligands, forming a chain running along the *a* axis with the dmbpy ligands hanging from the chain. A pair of symmetry-related chains are connected by Zn2 atoms, resulting in a one-dimensional ladder structure containing large windows [*ca* 10.245 (1) × 15.446 (2) Å^2^]. The ladders are extended into a three-dimensional supramolecular structure through hydrogen bonds (Table 1) (Desiraju, 2004) and π–π stacking interactions (Zang *et al.*, 2010), with centroid–centroid distances of 3.858 (2) and 3.911 (3) between the benzene rings and between the pyridine rings (Fig. 3).

### Experimental

All starting materials used in the synthesis were of analytical grade and obtained from commercial sources without further purification. The title compound was synthesized hydrothermally in a Teflon-lined stainless steel container by heating a mixture of benzene-1,3,5-tricarboxylic acid (0.011 g, 0.05 mmol), 5,5'-dimethyl-2,2'-bipyridine (0.009 g, 0.05 mmol), Zn(NO_3_)_2_.6H_2_O (0.015 g, 0.05 mmol) and NaOH (0.004 g, 0.1 mmol) in 7 ml of distilled water at 120°C for 3 d, and then cooled to room temperature. Washed with deionized water and dried, colorless block crystals of the title compound were obtained in 72% yield based on zinc.

### Refinement

H atoms were positioned geometrically and refined using a riding model, with C—H = 0.93 (aromatic) and 0.96 (methyl) Å and with *U*_iso_(H) = 1.2(1.5 for methyl)*U*_eq_(C). The approximate positions of the water H atoms were obtained from a difference Fourier map, then restrained to ideal configuration and fixed in the final stages of the refinement.

### Crystal data

**Table:**

[Zn_3_(C_9_H_3_O_6_)_2_(C_12_H_12_N_2_)_2_(H_2_O)_6_]·6H_2_O	*Z* = 1
*M**_r_* = 1195.00	*F*(000) = 616
Triclinic, *P*1	*D*_x_ = 1.608 Mg m^−^^3^
Hall symbol: -P 1	Mo *K*α radiation, λ = 0.71073 Å
*a* = 10.2454 (10) Å	Cell parameters from 3902 reflections
*b* = 10.5799 (10) Å	θ = 3.0–29.2°
*c* = 12.658 (1) Å	µ = 1.54 mm^−^^1^
α = 68.910 (8)°	*T* = 296 K
β = 74.848 (8)°	Block, colourless
γ = 81.834 (8)°	0.21 × 0.20 × 0.19 mm
*V* = 1233.90 (19) Å^3^	

### Data collection

**Table:**

Bruker APEXII CCD diffractometer	4330 independent reflections
Radiation source: fine-focus sealed tube	3426 reflections with *I* > 2σ(*I*)
graphite	*R*_int_ = 0.034
φ and ω scans	θ_max_ = 25.0°, θ_min_ = 3.0°
Absorption correction: multi-scan (*SADABS*; Bruker, 2001)	*h* = −12→11
*T*_min_ = 0.739, *T*_max_ = 0.759	*k* = −12→12
9149 measured reflections	*l* = −14→15

### Refinement

**Table:**

Refinement on *F*^2^	Primary atom site location: structure-invariant direct methods
Least-squares matrix: full	Secondary atom site location: difference Fourier map
*R*[*F*^2^ > 2σ(*F*^2^)] = 0.045	Hydrogen site location: inferred from neighbouring sites
*wR*(*F*^2^) = 0.124	H-atom parameters constrained
*S* = 1.01	*w* = 1/[σ^2^(*F*_o_^2^) + (0.0713*P*)^2^ + 0.3228*P*] where *P* = (*F*_o_^2^ + 2*F*_c_^2^)/3
4330 reflections	(Δ/σ)_max_ < 0.001
336 parameters	Δρ_max_ = 0.74 e Å^−^^3^
12 restraints	Δρ_min_ = −0.59 e Å^−^^3^

### Fractional atomic coordinates and isotropic or equivalent isotropic displacement parameters (Å^2^)

**Table:**

	*x*	*y*	*z*	*U*_iso_*/*U*_eq_	Occ. (<1)
Zn1	0.12533 (4)	0.26423 (4)	0.27950 (4)	0.02392 (16)	
Zn2	0.5000	−0.5000	0.5000	0.0307 (2)	
O1	0.2755 (2)	0.1223 (3)	0.3318 (2)	0.0318 (7)	
O2	0.3634 (3)	0.3178 (3)	0.2870 (3)	0.0402 (7)	
O3	0.8425 (3)	0.3214 (2)	0.3035 (2)	0.0308 (7)	
O4	0.9762 (2)	0.1367 (2)	0.3164 (2)	0.0291 (6)	
O5	0.7603 (3)	−0.3095 (2)	0.4380 (3)	0.0328 (7)	
O6	0.5455 (3)	−0.3002 (3)	0.4271 (3)	0.0361 (7)	
O1W	0.0603 (3)	0.3070 (3)	0.4344 (2)	0.0333 (7)	
H1WA	0.1250	0.3022	0.4656	0.050*	
H1WB	−0.0007	0.2560	0.4821	0.050*	
O2W	0.4783 (3)	−0.4920 (3)	0.3334 (3)	0.0432 (8)	
H2WA	0.4387	−0.5581	0.3356	0.065*	
H2WB	0.4504	−0.4322	0.2770	0.065*	
O3W	0.7074 (3)	−0.5649 (3)	0.4677 (3)	0.0408 (8)	
H3WB	0.7155	−0.6398	0.4555	0.061*	
H3WC	0.7508	−0.5056	0.4082	0.061*	
O4W	0.4365 (14)	0.6435 (16)	0.0453 (12)	0.117 (3)	0.408 (9)
H4WA	0.4059	0.6795	0.1072	0.175*	0.408 (9)
H4WB	0.4578	0.5864	0.0534	0.175*	0.408 (9)
O4W'	0.4797 (10)	0.5541 (10)	0.0114 (9)	0.117 (3)	0.592 (9)
H4WD	0.4529	0.4924	0.0792	0.175*	0.592 (9)
H4WE	0.4258	0.6206	−0.0140	0.175*	0.592 (9)
O5W	0.3621 (5)	0.7702 (6)	0.2466 (6)	0.142 (2)	
H5WA	0.2969	0.7985	0.2914	0.213*	
H5WC	0.4226	0.7649	0.2885	0.213*	
O6W	0.1335 (5)	0.8711 (5)	0.4028 (4)	0.1048 (18)	
H6WB	0.1775	0.9325	0.4047	0.157*	
H6WD	0.0961	0.9389	0.3584	0.157*	
N1	0.1983 (3)	0.2550 (3)	0.1050 (3)	0.0302 (8)	
N2	0.1122 (3)	0.4690 (3)	0.1716 (3)	0.0269 (7)	
C1	0.3732 (4)	0.1913 (4)	0.3168 (3)	0.0247 (8)	
C2	0.8611 (4)	0.1978 (4)	0.3186 (3)	0.0207 (8)	
C3	0.6509 (4)	−0.2463 (4)	0.4204 (3)	0.0248 (8)	
C4	0.5062 (3)	0.1165 (4)	0.3354 (3)	0.0216 (8)	
C5	0.6183 (3)	0.1867 (4)	0.3203 (3)	0.0235 (8)	
H5	0.6110	0.2808	0.2988	0.028*	
C6	0.7415 (3)	0.1176 (3)	0.3371 (3)	0.0207 (8)	
C7	0.7516 (3)	−0.0216 (3)	0.3692 (3)	0.0201 (8)	
H7	0.8338	−0.0679	0.3806	0.024*	
C8	0.6407 (4)	−0.0943 (3)	0.3851 (3)	0.0215 (8)	
C9	0.5189 (4)	−0.0233 (4)	0.3676 (3)	0.0225 (8)	
H9	0.4443	−0.0708	0.3777	0.027*	
C10	0.2447 (4)	0.1427 (4)	0.0791 (4)	0.0375 (10)	
H10	0.2463	0.0610	0.1400	0.045*	
C11	0.2910 (4)	0.1418 (5)	−0.0342 (4)	0.0412 (11)	
C12	0.3435 (6)	0.0116 (5)	−0.0579 (5)	0.0621 (15)	
H12A	0.3412	−0.0615	0.0144	0.093*	
H12B	0.4349	0.0210	−0.1032	0.093*	
H12C	0.2876	−0.0075	−0.1000	0.093*	
C13	0.2863 (5)	0.2651 (5)	−0.1217 (4)	0.0447 (11)	
H13	0.3157	0.2694	−0.1988	0.054*	
C14	0.2389 (5)	0.3818 (5)	−0.0967 (4)	0.0419 (11)	
H14	0.2364	0.4645	−0.1564	0.050*	
C15	0.1949 (4)	0.3746 (4)	0.0188 (3)	0.0304 (9)	
C16	0.1458 (4)	0.4930 (4)	0.0569 (4)	0.0287 (9)	
C17	0.1358 (4)	0.6233 (4)	−0.0215 (4)	0.0386 (11)	
H17	0.1577	0.6387	−0.1010	0.046*	
C18	0.0930 (5)	0.7297 (4)	0.0198 (4)	0.0443 (12)	
H18	0.0871	0.8175	−0.0322	0.053*	
C19	0.0590 (4)	0.7070 (4)	0.1378 (4)	0.0381 (11)	
C20	0.0129 (5)	0.8197 (5)	0.1863 (5)	0.0533 (13)	
H20A	0.0763	0.8258	0.2281	0.080*	
H20B	−0.0747	0.8018	0.2381	0.080*	
H20C	0.0073	0.9038	0.1240	0.080*	
C21	0.0697 (4)	0.5739 (4)	0.2107 (4)	0.0341 (10)	
H21	0.0465	0.5561	0.2906	0.041*	

### Atomic displacement parameters (Å^2^)

**Table:**

	*U*^11^	*U*^22^	*U*^33^	*U*^12^	*U*^13^	*U*^23^
Zn1	0.0188 (3)	0.0242 (3)	0.0267 (3)	−0.00367 (17)	−0.00609 (18)	−0.00453 (19)
Zn2	0.0273 (4)	0.0215 (3)	0.0435 (4)	−0.0059 (3)	−0.0063 (3)	−0.0107 (3)
O1	0.0175 (14)	0.0356 (15)	0.0417 (18)	−0.0021 (11)	−0.0126 (12)	−0.0080 (13)
O2	0.0321 (17)	0.0315 (16)	0.059 (2)	0.0060 (12)	−0.0200 (15)	−0.0145 (14)
O3	0.0290 (15)	0.0192 (14)	0.0441 (18)	−0.0061 (11)	−0.0087 (13)	−0.0086 (12)
O4	0.0138 (13)	0.0267 (14)	0.0460 (18)	−0.0001 (11)	−0.0073 (12)	−0.0112 (13)
O5	0.0298 (16)	0.0214 (14)	0.0508 (19)	0.0007 (11)	−0.0178 (14)	−0.0111 (13)
O6	0.0268 (15)	0.0224 (14)	0.062 (2)	−0.0077 (11)	−0.0145 (14)	−0.0120 (14)
O1W	0.0301 (15)	0.0433 (17)	0.0287 (16)	−0.0092 (12)	−0.0089 (12)	−0.0104 (13)
O2W	0.055 (2)	0.0332 (16)	0.046 (2)	−0.0066 (14)	−0.0158 (16)	−0.0136 (14)
O3W	0.0314 (16)	0.0288 (15)	0.062 (2)	−0.0021 (12)	−0.0040 (14)	−0.0191 (15)
O4W	0.113 (3)	0.119 (3)	0.118 (3)	−0.0102 (14)	−0.0282 (15)	−0.0372 (15)
O4W'	0.113 (3)	0.119 (3)	0.118 (3)	−0.0102 (14)	−0.0282 (15)	−0.0372 (15)
O5W	0.101 (4)	0.110 (4)	0.181 (6)	0.005 (3)	−0.051 (4)	0.001 (4)
O6W	0.134 (4)	0.123 (4)	0.060 (3)	−0.088 (3)	−0.015 (3)	−0.010 (3)
N1	0.0326 (19)	0.0304 (18)	0.0263 (19)	−0.0060 (14)	−0.0052 (15)	−0.0074 (15)
N2	0.0208 (17)	0.0305 (18)	0.028 (2)	−0.0039 (13)	−0.0064 (14)	−0.0063 (15)
C1	0.0166 (19)	0.034 (2)	0.024 (2)	0.0013 (16)	−0.0068 (15)	−0.0099 (17)
C2	0.021 (2)	0.0235 (19)	0.0158 (19)	−0.0072 (15)	−0.0019 (15)	−0.0034 (15)
C3	0.029 (2)	0.0240 (19)	0.023 (2)	−0.0083 (17)	−0.0042 (16)	−0.0090 (16)
C4	0.0177 (19)	0.029 (2)	0.018 (2)	−0.0007 (15)	−0.0030 (15)	−0.0088 (16)
C5	0.0210 (19)	0.0210 (18)	0.027 (2)	−0.0016 (15)	−0.0076 (16)	−0.0039 (16)
C6	0.0172 (18)	0.0230 (18)	0.023 (2)	−0.0042 (14)	−0.0044 (15)	−0.0075 (16)
C7	0.0162 (18)	0.0193 (17)	0.023 (2)	−0.0018 (14)	−0.0043 (15)	−0.0054 (15)
C8	0.024 (2)	0.0216 (18)	0.020 (2)	−0.0039 (15)	−0.0046 (15)	−0.0082 (16)
C9	0.0166 (19)	0.0274 (19)	0.024 (2)	−0.0048 (15)	−0.0049 (15)	−0.0085 (16)
C10	0.044 (3)	0.032 (2)	0.035 (3)	−0.0078 (19)	−0.009 (2)	−0.008 (2)
C11	0.040 (3)	0.051 (3)	0.039 (3)	−0.005 (2)	−0.007 (2)	−0.023 (2)
C12	0.077 (4)	0.057 (3)	0.058 (4)	0.001 (3)	−0.007 (3)	−0.034 (3)
C13	0.050 (3)	0.057 (3)	0.029 (3)	−0.008 (2)	−0.007 (2)	−0.017 (2)
C14	0.050 (3)	0.044 (3)	0.028 (3)	−0.008 (2)	−0.008 (2)	−0.006 (2)
C15	0.025 (2)	0.036 (2)	0.031 (2)	−0.0059 (17)	−0.0084 (18)	−0.0081 (19)
C16	0.025 (2)	0.029 (2)	0.033 (3)	−0.0058 (16)	−0.0085 (18)	−0.0090 (18)
C17	0.050 (3)	0.033 (2)	0.025 (2)	−0.005 (2)	−0.009 (2)	0.0014 (19)
C18	0.049 (3)	0.030 (2)	0.045 (3)	−0.003 (2)	−0.017 (2)	0.002 (2)
C19	0.031 (2)	0.031 (2)	0.050 (3)	0.0001 (18)	−0.012 (2)	−0.011 (2)
C20	0.056 (3)	0.040 (3)	0.063 (4)	0.001 (2)	−0.010 (3)	−0.020 (3)
C21	0.033 (2)	0.033 (2)	0.036 (3)	−0.0050 (18)	−0.0079 (19)	−0.0092 (19)

### Geometric parameters (Å, °)

**Table:**

Zn1—O4^i^	2.033 (2)	N2—C16	1.338 (5)
Zn1—O1	2.055 (3)	N2—C21	1.346 (5)
Zn1—O1W	2.089 (3)	C1—C4	1.505 (5)
Zn1—N2	2.112 (3)	C2—C6	1.509 (5)
Zn1—N1	2.172 (3)	C3—C8	1.502 (5)
Zn2—O6^ii^	2.047 (2)	C4—C9	1.382 (5)
Zn2—O6	2.047 (2)	C4—C5	1.390 (5)
Zn2—O3W^ii^	2.119 (3)	C5—C6	1.393 (5)
Zn2—O3W	2.119 (3)	C5—H5	0.9300
Zn2—O2W	2.148 (3)	C6—C7	1.375 (5)
Zn2—O2W^ii^	2.148 (3)	C7—C8	1.393 (5)
O1—C1	1.256 (4)	C7—H7	0.9300
O2—C1	1.250 (5)	C8—C9	1.389 (5)
O3—C2	1.246 (4)	C9—H9	0.9300
O4—C2	1.260 (4)	C10—C11	1.391 (6)
O4—Zn1^iii^	2.033 (2)	C10—H10	0.9300
O5—C3	1.250 (4)	C11—C13	1.378 (6)
O6—C3	1.260 (4)	C11—C12	1.510 (7)
O1W—H1WA	0.8433	C12—H12A	0.9600
O1W—H1WB	0.8428	C12—H12B	0.9600
O2W—H2WA	0.8457	C12—H12C	0.9600
O2W—H2WB	0.8499	C13—C14	1.375 (7)
O3W—H3WB	0.8500	C13—H13	0.9300
O3W—H3WC	0.8500	C14—C15	1.390 (6)
O4W—H4WA	0.9537	C14—H14	0.9300
O4W—H4WB	0.5942	C15—C16	1.477 (6)
O4W—H4WE	0.9037	C16—C17	1.387 (5)
O4W'—O4W'^iv^	1.27 (2)	C17—C18	1.377 (6)
O4W'—H4WB	0.7048	C17—H17	0.9300
O4W'—H4WD	0.8736	C18—C19	1.380 (6)
O4W'—H4WE	0.8501	C18—H18	0.9300
O5W—H5WA	0.8514	C19—C21	1.386 (6)
O5W—H5WC	0.9000	C19—C20	1.496 (6)
O6W—H6WB	0.8520	C20—H20A	0.9599
O6W—H6WD	0.8499	C20—H20B	0.9600
N1—C10	1.335 (5)	C20—H20C	0.9601
N1—C15	1.344 (5)	C21—H21	0.9300
			
O4^i^—Zn1—O1	98.75 (10)	O6—C3—C8	115.5 (3)
O4^i^—Zn1—O1W	95.22 (11)	C9—C4—C5	118.9 (3)
O1—Zn1—O1W	95.94 (11)	C9—C4—C1	120.4 (3)
O4^i^—Zn1—N2	121.50 (11)	C5—C4—C1	120.7 (3)
O1—Zn1—N2	137.13 (11)	C4—C5—C6	120.7 (3)
O1W—Zn1—N2	94.52 (12)	C4—C5—H5	119.7
O4^i^—Zn1—N1	91.75 (12)	C6—C5—H5	119.7
O1—Zn1—N1	89.17 (12)	C7—C6—C5	119.4 (3)
O1W—Zn1—N1	170.62 (11)	C7—C6—C2	121.7 (3)
N2—Zn1—N1	76.46 (13)	C5—C6—C2	119.0 (3)
O6^ii^—Zn2—O6	180.00 (15)	C6—C7—C8	121.1 (3)
O6^ii^—Zn2—O3W^ii^	91.78 (11)	C6—C7—H7	119.4
O6—Zn2—O3W^ii^	88.22 (11)	C8—C7—H7	119.4
O6^ii^—Zn2—O3W	88.22 (11)	C9—C8—C7	118.6 (3)
O6—Zn2—O3W	91.78 (11)	C9—C8—C3	120.0 (3)
O3W^ii^—Zn2—O3W	180.0	C7—C8—C3	121.4 (3)
O6^ii^—Zn2—O2W	91.61 (11)	C4—C9—C8	121.4 (3)
O6—Zn2—O2W	88.39 (11)	C4—C9—H9	119.3
O3W^ii^—Zn2—O2W	86.71 (12)	C8—C9—H9	119.3
O3W—Zn2—O2W	93.29 (12)	N1—C10—C11	123.4 (4)
O6^ii^—Zn2—O2W^ii^	88.39 (11)	N1—C10—H10	118.3
O6—Zn2—O2W^ii^	91.61 (11)	C11—C10—H10	118.3
O3W^ii^—Zn2—O2W^ii^	93.29 (12)	C13—C11—C10	116.4 (4)
O3W—Zn2—O2W^ii^	86.71 (12)	C13—C11—C12	122.7 (4)
O2W—Zn2—O2W^ii^	180.0	C10—C11—C12	120.9 (4)
C1—O1—Zn1	104.3 (2)	C11—C12—H12A	109.5
C2—O4—Zn1^iii^	112.2 (2)	C11—C12—H12B	109.5
C3—O6—Zn2	129.0 (2)	H12A—C12—H12B	109.5
Zn1—O1W—H1WA	111.8	C11—C12—H12C	109.5
Zn1—O1W—H1WB	112.0	H12A—C12—H12C	109.5
H1WA—O1W—H1WB	110.0	H12B—C12—H12C	109.5
Zn2—O2W—H2WA	114.2	C14—C13—C11	121.1 (4)
Zn2—O2W—H2WB	135.2	C14—C13—H13	119.5
H2WA—O2W—H2WB	97.6	C11—C13—H13	119.5
Zn2—O3W—H3WB	109.3	C13—C14—C15	119.2 (4)
Zn2—O3W—H3WC	109.3	C13—C14—H14	120.4
H3WB—O3W—H3WC	109.5	C15—C14—H14	120.4
H4WA—O4W—H4WB	121.9	N1—C15—C14	120.4 (4)
H4WA—O4W—H4WE	153.9	N1—C15—C16	115.2 (4)
H4WB—O4W—H4WE	73.1	C14—C15—C16	124.3 (4)
O4W'^iv^—O4W'—H4WB	148.4	N2—C16—C17	121.1 (4)
O4W'^iv^—O4W'—H4WD	78.5	N2—C16—C15	116.7 (3)
H4WB—O4W'—H4WD	71.6	C17—C16—C15	122.3 (4)
O4W'^iv^—O4W'—H4WE	133.7	C18—C17—C16	119.3 (4)
H4WB—O4W'—H4WE	72.4	C18—C17—H17	120.4
H4WD—O4W'—H4WE	119.7	C16—C17—H17	120.4
H5WA—O5W—H5WC	94.0	C17—C18—C19	120.5 (4)
H6WB—O6W—H6WD	82.4	C17—C18—H18	119.8
C10—N1—C15	119.6 (4)	C19—C18—H18	119.8
C10—N1—Zn1	125.4 (3)	C18—C19—C21	116.9 (4)
C15—N1—Zn1	115.0 (3)	C18—C19—C20	122.2 (4)
C16—N2—C21	119.0 (3)	C21—C19—C20	120.8 (4)
C16—N2—Zn1	116.5 (3)	C19—C20—H20A	109.5
C21—N2—Zn1	124.5 (3)	C19—C20—H20B	109.4
O2—C1—O1	122.5 (3)	H20A—C20—H20B	109.5
O2—C1—C4	119.7 (3)	C19—C20—H20C	109.5
O1—C1—C4	117.8 (3)	H20A—C20—H20C	109.5
O3—C2—O4	123.0 (3)	H20B—C20—H20C	109.5
O3—C2—C6	118.8 (3)	N2—C21—C19	123.2 (4)
O4—C2—C6	118.2 (3)	N2—C21—H21	118.4
O5—C3—O6	125.0 (3)	C19—C21—H21	118.4
O5—C3—C8	119.5 (3)		
			
O4^i^—Zn1—O1—C1	−177.5 (2)	C2—C6—C7—C8	−179.4 (3)
O1W—Zn1—O1—C1	−81.3 (3)	C6—C7—C8—C9	0.1 (6)
N2—Zn1—O1—C1	22.0 (3)	C6—C7—C8—C3	−179.9 (3)
N1—Zn1—O1—C1	90.9 (3)	O5—C3—C8—C9	−178.7 (4)
O3W^ii^—Zn2—O6—C3	152.0 (4)	O6—C3—C8—C9	3.0 (5)
O3W—Zn2—O6—C3	−28.0 (4)	O5—C3—C8—C7	1.3 (6)
O2W—Zn2—O6—C3	−121.2 (4)	O6—C3—C8—C7	−177.1 (3)
O2W^ii^—Zn2—O6—C3	58.8 (4)	C5—C4—C9—C8	0.1 (6)
O4^i^—Zn1—N1—C10	−60.6 (3)	C1—C4—C9—C8	179.9 (3)
O1—Zn1—N1—C10	38.1 (3)	C7—C8—C9—C4	−0.2 (6)
N2—Zn1—N1—C10	177.4 (3)	C3—C8—C9—C4	179.8 (3)
O4^i^—Zn1—N1—C15	119.6 (3)	C15—N1—C10—C11	0.0 (6)
O1—Zn1—N1—C15	−141.7 (3)	Zn1—N1—C10—C11	−179.8 (3)
N2—Zn1—N1—C15	−2.4 (3)	N1—C10—C11—C13	−0.3 (7)
O4^i^—Zn1—N2—C16	−80.7 (3)	N1—C10—C11—C12	179.5 (4)
O1—Zn1—N2—C16	76.6 (3)	C10—C11—C13—C14	0.3 (7)
O1W—Zn1—N2—C16	−179.7 (3)	C12—C11—C13—C14	−179.4 (5)
N1—Zn1—N2—C16	2.9 (3)	C11—C13—C14—C15	−0.1 (7)
O4^i^—Zn1—N2—C21	97.6 (3)	C10—N1—C15—C14	0.2 (6)
O1—Zn1—N2—C21	−105.1 (3)	Zn1—N1—C15—C14	180.0 (3)
O1W—Zn1—N2—C21	−1.3 (3)	C10—N1—C15—C16	−178.1 (3)
N1—Zn1—N2—C21	−178.7 (3)	Zn1—N1—C15—C16	1.7 (4)
Zn1—O1—C1—O2	6.0 (5)	C13—C14—C15—N1	−0.1 (6)
Zn1—O1—C1—C4	−173.7 (3)	C13—C14—C15—C16	178.0 (4)
Zn1^iii^—O4—C2—O3	−2.2 (5)	C21—N2—C16—C17	−0.9 (6)
Zn1^iii^—O4—C2—C6	176.1 (2)	Zn1—N2—C16—C17	177.5 (3)
Zn2—O6—C3—O5	14.6 (6)	C21—N2—C16—C15	178.5 (3)
Zn2—O6—C3—C8	−167.1 (2)	Zn1—N2—C16—C15	−3.0 (4)
O2—C1—C4—C9	−179.5 (4)	N1—C15—C16—N2	0.8 (5)
O1—C1—C4—C9	0.1 (5)	C14—C15—C16—N2	−177.4 (4)
O2—C1—C4—C5	0.3 (6)	N1—C15—C16—C17	−179.7 (4)
O1—C1—C4—C5	180.0 (3)	C14—C15—C16—C17	2.0 (6)
C9—C4—C5—C6	0.1 (6)	N2—C16—C17—C18	1.3 (6)
C1—C4—C5—C6	−179.8 (3)	C15—C16—C17—C18	−178.1 (4)
C4—C5—C6—C7	−0.1 (6)	C16—C17—C18—C19	−0.8 (7)
C4—C5—C6—C2	179.3 (3)	C17—C18—C19—C21	−0.1 (6)
O3—C2—C6—C7	−171.4 (3)	C17—C18—C19—C20	179.6 (4)
O4—C2—C6—C7	10.2 (5)	C16—N2—C21—C19	0.0 (6)
O3—C2—C6—C5	9.1 (5)	Zn1—N2—C21—C19	−178.4 (3)
O4—C2—C6—C5	−169.2 (3)	C18—C19—C21—N2	0.6 (6)
C5—C6—C7—C8	0.0 (6)	C20—C19—C21—N2	−179.2 (4)

Symmetry codes: (i) *x*−1, *y*, *z*; (ii) −*x*+1, −*y*−1, −*z*+1; (iii) *x*+1, *y*, *z*; (iv) −*x*+1, −*y*+1, −*z*.

### Hydrogen-bond geometry (Å, °)

**Table:**

*D*—H···*A*	*D*—H	H···*A*	*D*···*A*	*D*—H···*A*
O1W—H1WA···O5^v^	0.84	1.93	2.754 (4)	166
O1W—H1WB···O6W^vi^	0.84	1.96	2.805 (5)	176
O2W—H2WA···O2^vii^	0.85	1.95	2.768 (4)	162
O2W—H2WB···O5W^vii^	0.85	2.14	2.806 (6)	135
O3W—H3WB···O3^vii^	0.85	2.16	2.740 (4)	125
O3W—H3WC···O5	0.85	2.26	2.705 (4)	113
O4W—H4WA···O5W	0.95	2.22	3.166 (19)	173
O4W'—H4WD···O2	0.87	2.63	3.499 (11)	177
O5W—H5WA···O6W	0.85	2.14	2.987 (9)	179
O5W—H5WC···O6^viii^	0.90	2.28	3.151 (7)	164
O6W—H6WB···O1^viii^	0.85	2.16	2.947 (5)	154
O6W—H6WD···O4^ix^	0.85	2.23	3.019 (6)	154

Symmetry codes: (v) −*x*+1, −*y*, −*z*+1; (vi) −*x*, −*y*+1, −*z*+1; (vii) *x*, *y*−1, *z*; (viii) *x*, *y*+1, *z*; (ix) *x*−1, *y*+1, *z*.
